# Development of inflammation-induced hyperalgesia and allodynia is associated with the upregulation of extrasynaptic AMPA receptors in tonically firing lamina II dorsal horn neurons

**DOI:** 10.3389/fphys.2012.00391

**Published:** 2012-10-02

**Authors:** Olga Kopach, Viacheslav Viatchenko-Karpinski, Pavel Belan, Nana Voitenko

**Affiliations:** State Key Laboratory of Molecular and Cellular Biology, Bogomoletz Institute of PhysiologyKiev, Ukraine

**Keywords:** extrasynaptic AMPA receptors, development of inflammatory pain, substantia gelatinosa neurons, hyperalgesia, allodynia

## Abstract

Persistent peripheral inflammation changes AMPA receptor (AMPAR) trafficking in dorsal horn neurons by promoting internalization of GluR2-containing, Ca^2+^-impermeable AMPARs from the synapses and by increasing insertion of GluR1-containing, Ca^2+^-permeable AMPARs in extrasynaptic plasma membrane. These changes contribute to the maintenance of persistent inflammatory pain. However, much less is known about AMPAR trafficking during development of persistent inflammatory pain and direct studies of extrasynaptic AMPARs functioning during this period are still lacking. Using Complete Freund's adjuvant (CFA)-induced model of long-lasting peripheral inflammation, we showed that remarkable hyperalgesia and allodynia developes in 1–3 h after intraplantar CFA injection. By utilizing patch-clamp recording combined with Ca^2+^ imaging, we found a significant upregulation of extrasynaptic AMPARs in substantia gelatinosa (SG) neurons of the rat spinal cord 2–3 h after CFA injection. This upregulation was manifested as a robust increase in the amplitude of AMPAR-mediated currents 2–3 h post-CFA. These changes were observed specifically in SG neurons characterized by intrinsic tonic firing properties, but not in those that exhibited strong adaptation. Our results indicate that CFA-induced inflammation increases functional expression of extrasynaptic AMPARs in tonically firing SG neurons during development of pain hypersensitivity and that this increase may contribute to the development of peripheral persistent pain.

## Introduction

Persistent or chronic pain is a public health problem worldwide and may result from infection, inflammation, peripheral injury to tissue or nerve. Understanding the molecular mechanisms underlying development and maintenance of chronic pain ensures success of its treatment by developing alternative strategies, which target specific molecules precisely, and would therefore be of potential benefit in treatment or even prevention of persistent pain. Cumulative evidence demonstrates that altered AMPAR trafficking in dorsal horn neurons is required for the persistent inflammatory pain maintenance. However, a role of AMPARs in persistent inflammatory pain development is not completely understood. Besides, current studies in this field are predominantly concentrated on synaptic AMPARs, leaving functioning of extrasynaptic AMPARs population out of direct investigation.

α-amino-3-hydroxy-5-methyl-4-isoxazolepropionic acid receptors (AMPARs) expression level at extrasynaptic sites of neuronal plasma membrane is high throughout the central nervous system (CNS). Extrasynaptic AMPARs are localized within spines, dendrites, and somata (Malinow and Malenka, 2002; Bredt and Nicoll, 2003). They are mobile and rapidly move between the plasma membrane and intracellular compartments by exocytosis and endocytosis, and can migrate laterally to and from synaptic sites adjusting synaptic strength (Borgdorff and Choquet, 2002; Choquet and Triller, 2003; Adesnik et al., 2005; Cognet et al., 2006). Extrasynaptic AMPARs may also contribute to glutamatergic signaling at nonsynaptic locations. Glutamate, released from the presynaptic neurons and from astrocytes, can bath the extrasynaptic plasma membrane by the glutamate spillover and activates extrasynaptic AMPARs, thus strengthening glutamatergic transmission. During neuropathological conditions, when a primary afferent input is strong, glutamate spillover is excessive (Allan and Rothwell, 2001) and results in increased excitotoxic vulnerability and neurons injury (Kullmann, 2000; Allan and Rothwell, 2001; Weng et al., 2006).

The changes in AMPAR subunit trafficking, subunit composition, phosphorylation of AMPAR subunits or their interaction with partner proteins may contribute to spinal nociceptive transmission (Santos et al., 2009; Wang et al., 2010; He et al., 2011). In a spinal cord, AMPAR trafficking has been suggested as one of key mechanisms for central sensitization, a specific form of plasticity underlying induction and maintenance of pain (Hartmann et al., 2004; Park et al., 2008, 2009; Latremoliere and Woolf, 2009; Tao, 2010; Kopach et al., 2011). Recent studies demonstrated that altered AMPAR trafficking in dorsal horn neurons is causally linked to peripheral inflammatory pain maintenance (Hartmann et al., 2004; Katano et al., 2008; Park et al., 2009). Persistent peripheral inflammation promotes internalization of GluR2-containing, Ca^2+^-impermeable AMPARs from the synapses (Park et al., 2009) and insertion of GluR1-containing, Ca^2+^-permeable AMPARs at extrasynaptic membrane of lamina II dorsal horn neurons (Kopach et al., 2011) during inflammatory pain maintenance. However, much less is known about extrasynaptic AMPAR trafficking in dorsal horn neurons during development of inflammatory pain. So far as inflammation-induced peripheral hypersensitivity develops rapidly (2–3 h) after intraplantar complete Freund's adjuvant (CFA) injection and is comparable to one observed during the period of inflammatory pain maintenance (Zhang et al., 2003; Park et al., 2009), the same peripheral inflammatory insult may also potentially alter AMPAR trafficking in dorsal horn neurons when hypersensitivity is being developed. The altered AMPAR trafficking may in turn contribute to the observed hypersensitivity and strength pain development and maintenance.

In this work, we used electrophysiology combined with [Ca^2+^]_*i*_ imaging to study extrasynaptic AMPAR functioning in dorsal horn substantia gelatinosa (SG) neurons during the development period of peripheral inflammatory pain. We show that development of inflammation-induced hyperalgesia and allodynia is associated with the upregulation of extrasynaptic AMPA receptors (AMPARs) in specific subtype of lamina II dorsal horn neuron.

## Materials and methods

### Animal preparation

Male rats were housed in cages on a standard 12:12 h light/dark cycle. Water and food were available ad libitum until rats were transported to the laboratory for experiments. The animals were used in accordance with protocols that were approved by the Animal Care and Use Committee at the Bogomoletz Institute of Physiology and were consistent with the ethical guidelines of the National Institutes of Health and the International Association for the Study of Pain. All efforts were made to minimize animal suffering and to reduce the number of animals used.

### Experimental drugs

CFA was purchased from Sigma Chemical Co. (St. Louis, MO). Fura-2 was obtained from Invitrogen (Carlsbad, CA, USA). Tetrodotoxin (TTX) was obtained from Alomone Labs Ltd. (Jerusalem, Israel). NBQX, APV, AMPA, cyclothiazide (CTZ), bicuculline, and strychnine were purchased from Tocris Bioscience (Ellisville, MO).

### Induction of peripheral inflammation

To produce unilateral peripheral inflammation and nociceptive hypersensitivity, 100 μl of CFA (*Mycobacterium tuberculosis*) suspended in an oil-saline (1:1) emulsion was injected subcutaneously into the plantar side of one hind paw of the rats. Saline injection (0.9%; 100 μl) was used as a control.

### Behavioral testing

Animals were acclimatized to the experimental setup before the testing. The experimenters were blinded to the treatment groups during behavioral testing.

Paw withdrawal responses to thermal stimuli were measured in rats by using the Hargreaves technique (Hargreaves et al., 1988). For measurement of paw withdrawal responses to noxious heat stimuli, the animal was placed in a Plexiglas chamber on a glass plate located above a light box (Bioseb, Italy). Radiant heat was applied by focused infrared beam through a hole in the light box through the glass plate to the middle of the plantar surface of each hind paw. When the animal lifted its foot, the light beam was automatically turned off. The length of time between the start of the beam and the foot lift was defined as the paw withdrawal latency. Each trial was repeated five times at 5-min intervals for each paw. A cut-off time of 30 s was used to avoid tissue damage. Behavioral tests were performed before and after CFA injection.

To measure paw withdrawal responses to repeated mechanical stimuli, we used von Frey method. A rat was placed in a Plexiglas chamber on an elevated mesh screen and each von Frey monofilament (Bioseb, Italy) was applied to the hind paw for approximately 1–2 s. Trials of each hind paw were repeated 10 times at 1-min intervals. The occurrence of paw withdrawal in each of these trials was expressed as a percentage response frequency.

### Spinal cord slice preparation

Spinal cord slices were prepared from 18–21-day-old male rats subjected to saline or CFA injection as described previously (Voitenko et al., 2004; Kopach et al., 2011). Briefly, after rats were deeply anesthetized with an overdose of isoflurane, the L_4−5_ spinal segments were removed. Transverse slices (300 μm thick) were cut on a vibratome (Campden Instrument, UK) in an ice-cold solution that was continuously bubbled with 95% O_2_ and 5% CO_2_ and contained (in mM) 250 sucrose, 2 KCl, 1.2 NaH_2_PO_4_, 0.5 CaCl_2_, 7 MgCl_2_, 26 NaHCO_3_, and 11 glucose (pH 7.4). Slices were maintained at room temperature in a physiologic Krebs bicarbonate solution that contained (in mM) 125 NaCl, 2.5 KCl, 1.25 NaH_2_PO_4_, 2 CaCl_2_, 1 MgCl_2_, 26 NaHCO_3_, and 10 glucose (pH 7.4, osmolarity 310–320 mOsM) and was equilibrated with 95% O_2_ and 5% CO_2_.

### Simultaneous Ca^2+^ imaging and patch-clamp recording

Simultaneous Ca^2+^ imaging and whole-cell electrophysiologic recordings were obtained from SG neurons of the spinal L_4−5_ dorsal horn as described previously (Voitenko et al., 2004; Kopach et al., 2011). Briefly, the neurons were visually identified with a video microscope (Olympus, Japan). The patch pipettes with resistance of 6–10 MΩ were filled with an internal solution containing (in mM) 133 K-gluconate, 5 NaCl, 0.5 MgCl_2_, 10 HEPES-Na, 2 MgATP, 0.1 GTP-Na, and 0.2 fura-2 pentapotassium salt (pH 7.2, osmolarity 290 mOsM). The membrane potential of SG neurons was held at –60 mV by a patch-clamp amplifier PC-505B (Warner Instruments, Hamden, CT) and Digidata board 1320A (Molecular Devices, Union City, CA) controlled by pCLAMP 8.2 software (Axon Instruments, USA) in current or voltage-clamp mode. Only data from neurons that exhibited a resting membrane potential negative to –60 mV were included in the analysis.

Neurons were randomly chosen in the SG, but were mainly localized in its media-lateral part (Figure 3A). All SG neurons were categorized according to their discharge pattern in response to the series of 1-s current pulses, as described previously (Kopach et al., 2011).

To isolate AMPAR-mediated membrane current and associated increase in free calcium concentration in cytosol ([Ca^2+^]_*i*_), we performed recordings in the continuous presence of D-APV (50 μM), bicuculline (5 μM), and strychnine (2 μM) to block NMDA, GABA_A_, and glycine receptors, respectively. In addition, TTX (0.5 μM) and cadmium chloride (100 μM) were added to Krebs bicarbonate solution to block corresponding voltage-activated sodium and calcium channels. To prevent a desensitization of AMPARs during bath application of the agonist, we applied AMPA to the slices in the continuous presence of CTZ (20 μM). Typically, one neuron was studied per slice.

Fura-2 fluorescence from SG neurons, located between 50 and 100 μm below the surface of the slice, was measured by using a 60×, NA 0.9 water-immersion objective (Olympus, Japan) and a 12-bit cooled CCD camera and capturing board (Sensicam, PCO, Germany). Fluorescence intensity was measured at wavelengths >510 nm when excited at 380 and 340 nm by a PolyChrome IV monochromator (Till Photonics, Germany) using Imaging Workbench software (INDEC System, USA). Fura-2 fluorescence were measured in soma and dendrites of SG neurons and expressed as changes in the ratio of fluorescence at 340–380 nm. The background area closest to the areas of interest was subtracted for each wavelength. The amplitude of the AMPA-induced [Ca^2+^]_*i*_ transient was calculated as the difference between the fura-2 fluorescence ratio before AMPA application and the ratio at the maximum [Ca^2+^]_*i*_ rise during AMPARs activation.

### Statistical analysis

All data are presented as mean ± SEM with n referring to the number of cells analyzed. Student's *t*-tests (two-tailed unpaired) were used to determine statistically significant differences. A *p*-value of less than 0.05 was considered statistically significant.

## Results

### Development of CFA-induced peripheral hypersensitivity

**Figure 1:**
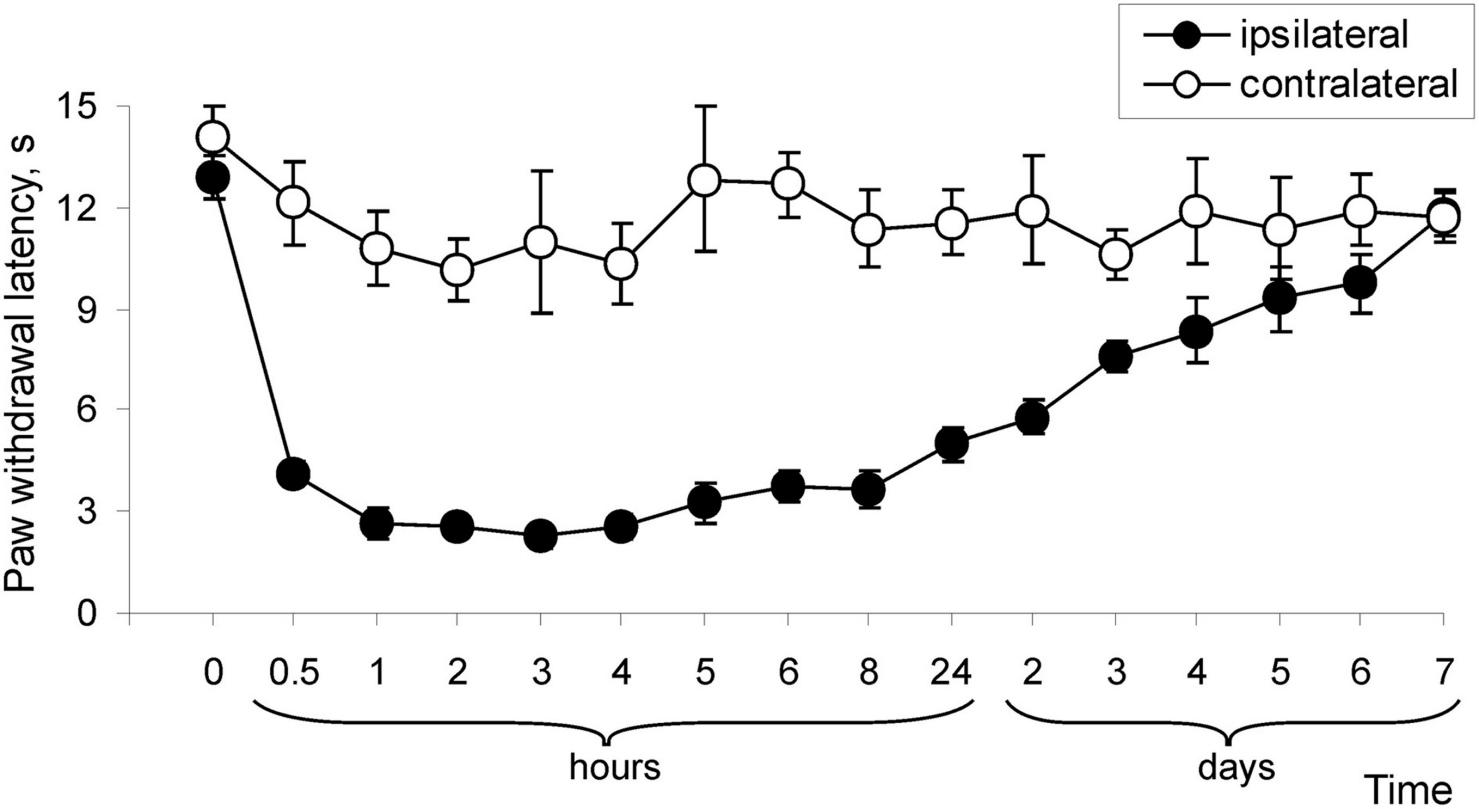
**Development of CFA-induced peripheral thermal hypersensitivity in rats.** CFA injection induced a decrease in paw withdrawal latency in response to thermal stimulation (*n* = 6/time point).

Our previous studies (Park et al., 2009) and those of others (Zhang et al., 2003; Park et al., 2008) show that subcutaneous injection of CFA into a hind paw led to development of robust hypersensitivity on the ipsilateral side. This inflammation-induced hypersensitivity reached a peak around 24 h after the injection of CFA and maintained for a few days, representing the maintenance period of persistent pain. Here, we examined how persistent pain develops. For this, we thoroughly measured paw withdrawal responses to both thermal and mechanical stimuli. before CFA injection, and then at 0.5, 1, 2, 3, 4, 5, 6, 7, and 8 h after CFA injection. Consistent with previous reports (Zhang et al., 2003), CFA injection produced a rapid development of thermal hypersensitivity, as evidenced by a decrease in ipsilateral paw withdrawal latency (Figure 1). This hypersensitivity was observed even at 0.5 h after CFA injection and further progressed in following 2–4 h (*n* = 6; *p* < 0.01; Figure 1). After 4–8 h, this hypersensitivity was maintained at the similar level for a few days after CFA injection, representing maintenance period of CFA-induced inflammatory pain (Figure 1).

**Figure 2:**
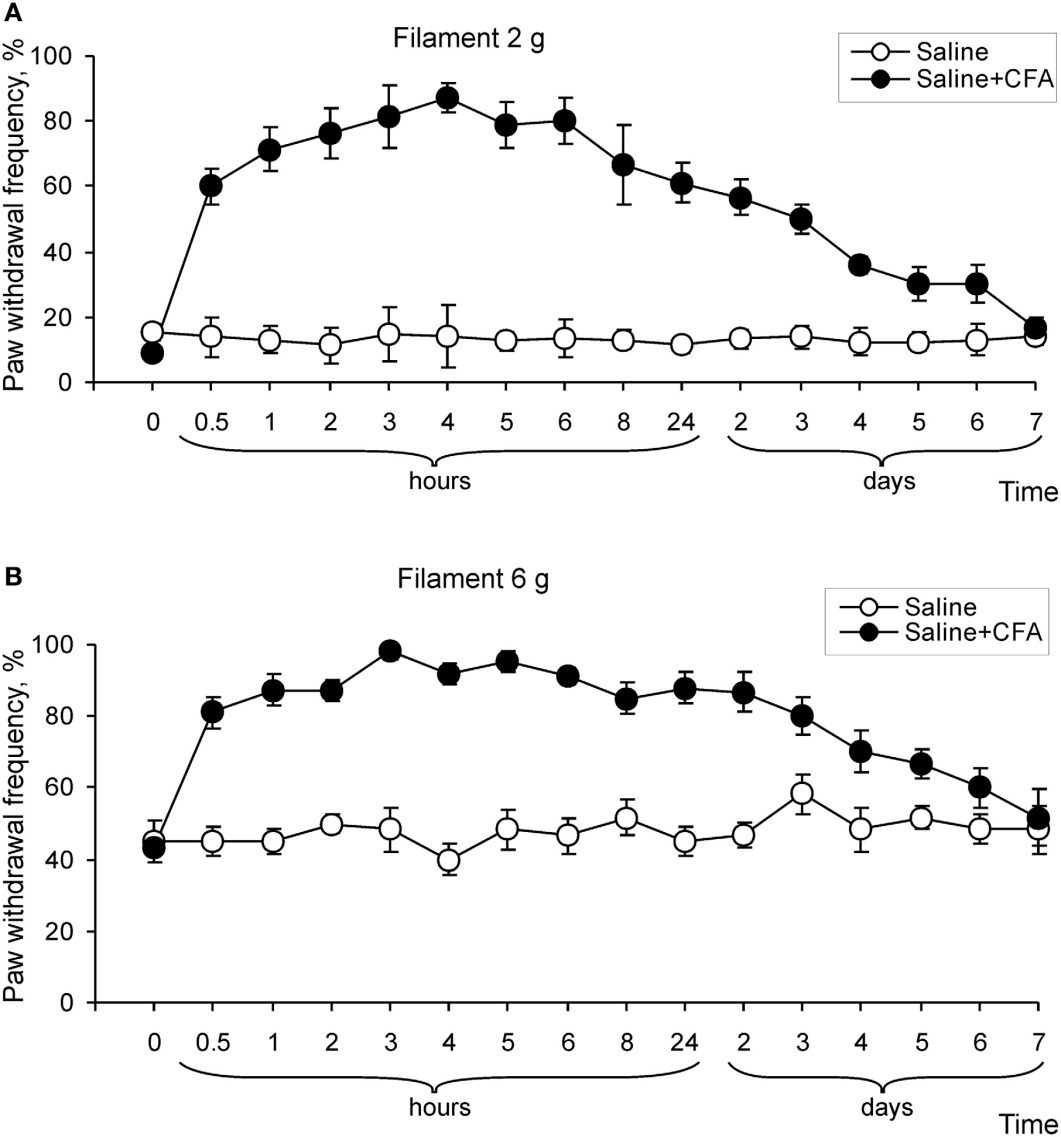
**Development of mechanical hypersensitivity after CFA injection. (A,B)** Paw withdrawal frequency in response to 2 g **(A)** and 6 g **(B)** von Frey filaments changed over time on the ipsilateral side after CFA, but not after saline injection into a hind paw (*n* > 6/time point for saline and CFA).

CFA (but not saline) injection led also to development of mechanical hypersensitivity that manifested as a marked increase in paw withdrawal frequencies in response to von Frey filaments applied to the injected hind paw (Figure 2). As with the paw withdrawal latency, a significant increase in paw withdrawal frequency started at 0.5 h after CFA injection and further progressed over 2–4 h (*n* = 8; *p* < 0.01), then was maintained for a few days (Figure 2). In addition, CFA injection significantly increased the paw withdrawal frequencies in response to innoxious (2 and 4 g) and noxious (6 and 8 g) von Frey filaments, indicating development of mechanical allodynia and hyperalgesia, respectively (*n* = 6; *p* < 0.01; Figures 2A,B; data for 4 and 8 g are not shown). These results indicate that remarkable thermal and mechanical hypersensitivities develop in a time interval of 1–6 h after CFA injection and these changes of peripheral sensitivity are comparable to the changes observed during the maintenance period of chronic inflammatory pain (1–7 days after CFA injection).

### CFA-induced inflammation upregulates extrasynaptic AMPARs in tonically firing SG neurons rather than in transient neurons

**Figure 3:**
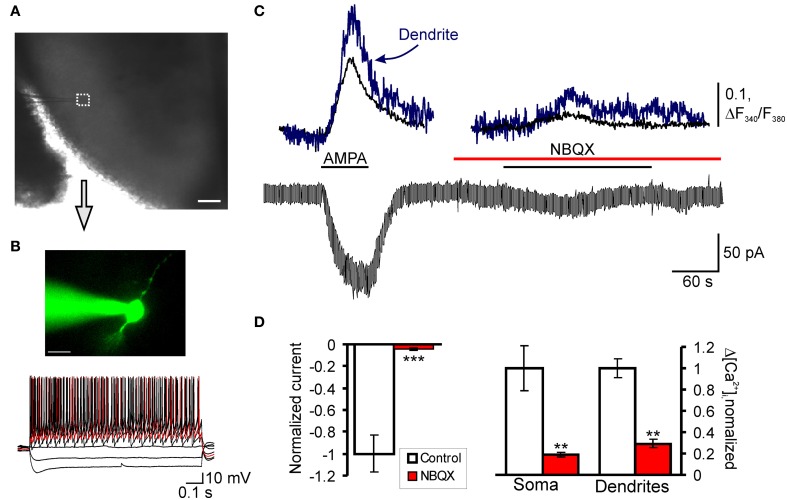
**AMPAR-mediated currents and associated [Ca^2+^]_*i*_ transients in SG neurons of spinal cord slices. (A)** Transmitted light image of patch electrode position (square) in the SG of a transverse spinal cord slice; scale bar = 200 μm. **(B)** A fluorescent image of SG neuron loaded with fura-2 (200 μM) and current-clamp recording of typical firing pattern for tonic neurons; scale bar = 20 μm. **(C)** Representative trace of a membrane current recorded from the soma in tonic neurons during AMPA bath application (5 μM) before and after pre-application of AMPAR antagonist NBQX (30 μM). **(D)** A statistical summary of the AMPA-induced current amplitudes (left graph) and [Ca^2+^]_*i*_ transients (right graph) in soma and dendrites of SG neurons before and after pre-application of AMPAR antagonists NBQX. ^**^p < 0.01, ^***^p < 0.001 versus control.

Previously we showed that CFA-induced peripheral inflammation substantially increases functional expression of Ca^2+^-permeable AMPARs in the extrasynaptic plasma membrane of lamina II dorsal horn neurons during the maintenance period of persistent inflammatory pain (Kopach et al., 2011) and altered AMPAR trafficking is necessary for the maintenance of inflammatory pain (Park et al., 2009). In this work, we studied whether CFA-induced peripheral inflammation affects extrasynaptic AMPARs functioning in these neurons during the development phase of persistent inflammatory pain. Because of our behavioral results and those of others (Zhang et al., 2003) have shown that CFA-induced robust thermal and mechanical hypersensitivities develop over 2–4 h after CFA injection, we chose the time point of 3 h after CFA injection to represent the development phase of CFA-induced persistent inflammatory pain. To directly evaluate CFA-induced changes in functional expression of extrasynaptic AMPARs during inflammatory pain development, we performed simultaneous recordings of membrane current and associated [Ca^2+^]_*i*_ transients produced by a selective agonist of AMPARs, AMPA, 3 h after saline or CFA injection (Figures 3A,B). We recorded AMPA-induced [Ca^2+^]_*i*_ transients in both the soma and dendrites of SG neurons.

Consistent with our previous reports (Voitenko et al., 2004; Kopach et al., 2011), bath application of AMPA (5 μM, 60 s) to the spinal cord slices evoked an inward current in all SG neurons at a holding potential of –60 mV. This current was characterized by a slow rising phase and subsequent plateau, reached within 20–60 seconds (Figure 3C). Although bath applied AMPA activates a total pool of plasma membrane AMPARs, the extrasynaptic receptors are the predominant contributor to AMPA-induced current because they are relatively more abundant in neuronal plasma membrane and have weaker desensitization than synaptic AMPARs (Guire et al., 2008; Arendt et al., 2010; Vizi et al., 2010). In addition, substantial differences in a rectification index and a sensitivity to polyamine blockers between AMPA-induced currents (Kopach et al., 2011) and AMPAR-mediated excitatory postsynaptic currents evoked by dorsal root stimulation in SG neurons (Katano et al., 2008; Vikman et al., 2008; Park et al., 2009) further confirm a major contribution of extrasynaptic receptors to AMPA-induced currents.

The AMPA-induced current was associated with a synchronous rise in [Ca^2+^]_*i*_ observed in both soma and dendrites of examined neurons (Figure 3C). The observed current and associated [Ca^2+^]_*i*_ transients were mediated by an activation of AMPARs, as a potent AMPAR antagonist NBQX (30 μM) almost completely inhibited both the current and [Ca^2+^]_*i*_ transients (Figures 3C,D). Other selective AMPAR antagonist, GYKI 52466, also inhibited both the current and associated [Ca^2+^]_*i*_ transients similar to NBQX (Kopach et al., 2011), further indicating an activation of AMPARs.

Taking into account that the population of SG neurons is not homogeneous and displays distinct immunohistochemical (Engelman et al., 1999), electrophysiological (Grudt and Perl, 2002) and functional properties (Graham et al., 2004), we divided all neurons accordingly to their firing pattern in a response to sustained depolarizing current. Furthermore, our previous results have shown that persistent peripheral inflammation changes extrasynaptic AMPAR trafficking specifically in subpopulation of SG neurons characterized by intrinsic tonic firing properties, but not in those that exhibited strong adaptation (Kopach et al., 2011). Therefore, we divided all SG neurons tested into two main groups: “tonic” and “transient.” Tonic neurons were those that supported continued discharge of action potentials during 1-s depolarizing inward current with increased frequency of discharge in a response to increased current intensity (Figure 3B). Transient neurons were those that exhibited a strong adaptation by generating short bursts of spikes or just a single spike regardless of depolarizing current intensity (Figure 5A).

**Figure 4:**
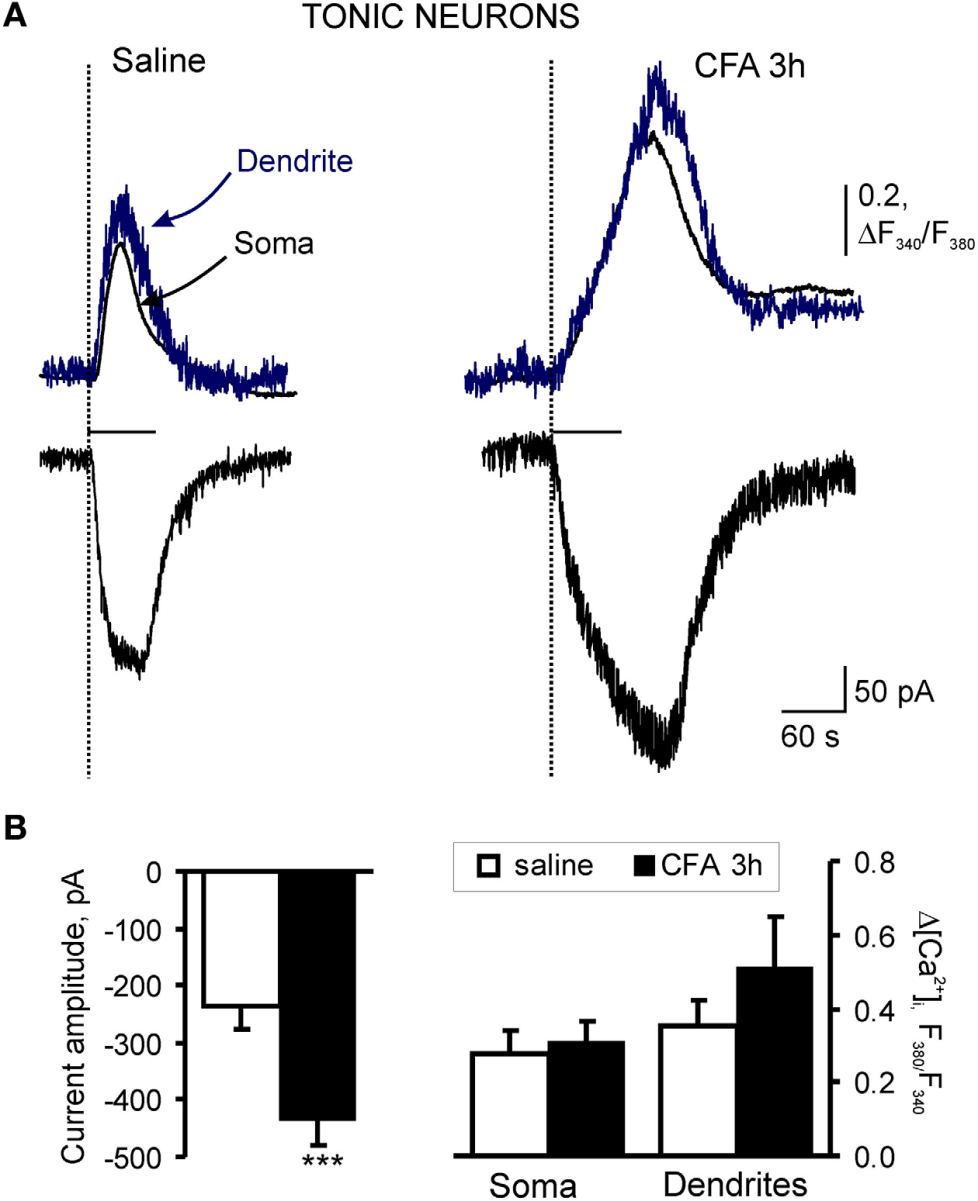
**Extrasynaptic AMPARs are markedly upregulated in tonic SG neurons during development of CFA-induced inflammatory pain. (A)** Representative examples of AMPA-induced membrane current (bottom traces) and associated [Ca^2+^]_*i*_ transients in soma (upper black traces) and dendrites (upper blue traces) in tonic neurons 3 h after saline (left traces) or CFA injection (right traces). **(B)** A statistical summary of current amplitudes (left graph) and [Ca^2+^]_*i*_ transients (right graph) in soma and dendrites of tonic SG neurons 3 h after saline or CFA injection. AMPA-induced currents were significantly increased during chronic pain development. Despite the tendency toward an increase in [Ca^2+^]_*i*_ transients in SG neurons 3 h after CFA injection, no significant change in their amplitudes was observed. ^***^p < 0.001 versus the saline-treated group.

**Figure 5:**
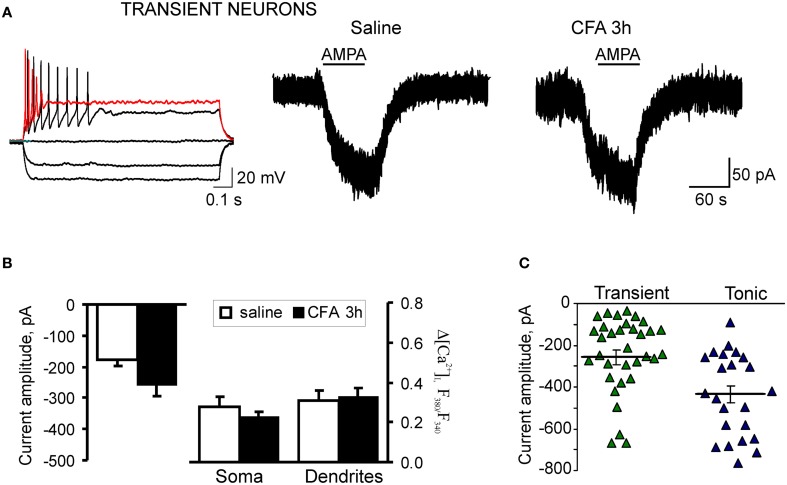
**Extrasynaptic AMPARs functioning is not altered in the transient SG neurons during development of CFA-induced inflammatory pain. (A)** Typical firing pattern (left traces) and representative examples of AMPA-induced somatic currents (right traces) in the transient SG neurons 3 h after saline (control) or CFA injection. **(B)** A statistical summary of the current amplitudes (left graphs) and [Ca^2+^]_*i*_ transients (right graphs) in soma and dendrites of transient SG neurons 3 h after injection of saline or CFA. No significant difference was found in all measured parameters. **(C)** The scatter plot illustrates a spread in AMPA-induced currents in the tonic (blue) and transient (green) neurons 3 h after CFA injection. Difference in the amplitudes of AMPA-induced currents between the transient and tonic SG neurons was significant in the CFA-inflamed group (*p* < 0.01), but not significant 3 h post-saline (*p* > 0.05).

In tonically firing SG neurons, the average amplitude of AMPA-induced currents comprised 236 ± 39 pA (*n* = 23) in the saline-treated group; the amplitude of associated [Ca^2+^]_*i*_ transients, expressed as an increase in the ratio of fura-2 fluorescence at 340–380 nm, ΔR (see “Materials and Methods”), were 0.47 ± 0.10 (*n* = 21) for soma and 0.60 ± 0.12 (*n* = 14) for dendrites (Figures 4A,B). CFA-induced inflammation significantly increased the amplitude of AMPA-induced current in the tonic SG neurons 3 h after CFA injection (Figure 5C). In particular, the average amplitude of AMPA-induced current was −236 ± 39 pA (*n* = 23) in 3 h post-saline group, but −437 ± 41 pA (*n* = 23; *p* < 0.001) in 3 h post-CFA group (Figure 4B). CFA-induced inflammation led to a tendency toward an increase in the amplitude of associated [Ca^2+^]_*i*_ transients in soma and dendrites of tonic SG neurons 3 h after injection (Figures 4A,B). In soma, the average amplitudes of AMPA-induced [Ca^2+^]_*i*_ transients were 0.47 ± 0.10 (*n* = 21) vs 0.54 ± 0.10 (*n* = 20) in the post-saline and post-CFA groups, respectively (Figure 4B). In dendrites, the average amplitudes of [Ca^2+^]_*i*_ transients were 0.60 ± 0.12 (*n* = 14) 3 h after saline vs 0.84 ± 0.23 (*n* = 15) 3 h after CFA (Figure 4B). However, these changes were not significant (*p* > 0.05).

These findings indicate that CFA-induced peripheral inflammation significantly upregulates functional expression of extrasynaptic AMPARs in tonically firing SG neurons during the development period of inflammatory pain.

CFA-induced inflammation did not significantly change the AMPA-induced current and associated [Ca^2+^]_*i*_ transients in the transient subtype of SG neurons during the development period of inflammatory pain. In the saline-treated group, average amplitude of AMPA-induced current was −177 ± 22 pA (*n* = 24) in the transient SG neurons that was not significantly different from that in the tonic neurons (*p* > 0.05). In the transient SG neurons from the CFA-treated group, the current amplitude was −259 ± 35 pA at 3 h after CFA injection (*n* = 34; *p* > 0.05 compared to the saline-treated group; Figures 5A–C). No significant differences were observed also in the average amplitudes of AMPA-induced [Ca^2+^]_*i*_ transients in soma and dendrites of transient SG neurons between the saline- and CFA-treated groups 3 h after injection. In soma, average amplitudes of [Ca^2+^]_*i*_ transients were: 0.48 ± 0.09 (*n* = 22) and 0.39 ± 0.04 (*n* = 31) for the saline- and CFA-treated groups, respectively (*p* > 0.05; Figure 5B). In dendrites, average amplitudes of [Ca^2+^]_*i*_ transients were 0.52 ± 0.09 (*n* = 18) for the saline-treated and 0.54 ± 0.09 (*n* = 18) for the CFA-treated groups 3 h after injection (*p* > 0.05; Figure 5B). These results demonstrate that in the transient type of SG neurons extrasynaptic AMPARs functioning is not significantly altered during development of CFA-induced inflammatory pain.

## Discussion

In this work, we have demonstrated that extrasynaptic AMPARs are significantly upregulated in tonically firing dorsal horn SG neurons during the development of persistent inflammatory pain. This upregulation of AMPARs in dorsal horn neurons is associated with inflammation-induced hyperalgesia and allodynia and might contribute to the development and maintenance of persistent inflammatory pain.

Our findings and those of others (Zhang et al., 2003; Park et al., 2009) show that CFA-induced peripheral inflammation produces rapid development of peripheral hyperalgesia and allodynia (2–3 h after CFA injection) that represents developmental period of persistent inflammatory pain, whereas pain hypersensitivities observed during 1–7 d post-CFA reflects the maintenance phase of inflammatory pain (Figures 1 and 2). Spinal cord dorsal horn sensitization is considered to be a central mechanism that underlies the induction and maintenance of pain hypersensitivities (Woolf and Salter, 2000; Latremoliere and Woolf, 2009; Wang et al., 2010). Central sensitization produces a substantial prolongation and enhancement of responses to both noxious and innocuous stimuli (hyperalgesia and allodynia) and is a specific form of synaptic plasticity in spinal cord, which depends crucially on recruitment of glutamate receptors (Woolf and Salter, 2000; Latremoliere and Woolf, 2009; Wang et al., 2010). Activation of particularly NMDA receptors and AMPARs in dorsal horn neurons is required for the development and maintenance of spinal sensitization and hyperalgesia (Woolf and Salter, 2000; Hartmann et al., 2004; Katano et al., 2008; Vikman et al., 2008; Latremoliere and Woolf, 2009; Park et al., 2009).

Previously we and others showed that persistent inflammation produces a switch of Ca^2+^-impermeable to Ca^2+^-permeable AMPAR-mediated neurotransmission at synapses in dorsal horn during the maintenance of inflammatory pain (Katano et al., 2008; Vikman et al., 2008; Park et al., 2009) and these changes are necessary for the peripheral inflammatory pain maintenance (Park et al., 2009). Peripheral inflammatory insult also increases functional expression of extrasynaptic Ca^2+^-permeable AMPARs in the tonically firing SG neurons during the maintenance period of inflammatory pain, as we reported recently (Kopach et al., 2011). Our current work illustrates that CFA-induced inflammation substantially upregulates extrasynaptic AMPARs in tonically firing SG neurons during the inflammatory pain development (3 h after CFA injection). Our electrophysiological results suggest that the number of extrasynaptic AMPARs is substantially increased in the SG neurons during development of CFA-induced peripheral hyperalgesia and allodynia. This upregulation of AMPARs may contribute to the observed hypersensitivities during pain development, as altered AMPAR trafficking is necessary for the persistent pain maintenance (Park et al., 2009). In contrast to the maintenance of inflammatory pain, AMPAR-mediated Ca^2+^ signaling in the tonic firing SG neurons was not significantly changed during the inflammatory pain development. We suggest that the upregulation of extrasynaptic AMPARs during pain development may be mediated mainly by an increase in GluR2-containing, Ca^2+^-impermeable AMPARs followed by a subsequent upregulation of GluR2-lacking, Ca^2+^-permeable extrasynaptic AMPARs, which was observed during the maintenance period of persistent inflammation (Kopach et al., 2011). Phosphorylation of AMPAR subunits may underlie the observed CFA-induced upregulation of extrasynaptic AMPARs in dorsal horn SG neurons during inflammatory pain development. As we have demonstrated previously, CFA injection significantly changes the level of GluR2 phosphorylation 2 h after CFA injection (Park et al., 2009), supporting our current electrophysiological results. However, the precise mechanisms of AMPARs regulation are not completely clear because of their complexity (Santos et al., 2009; Wang et al., 2010; He et al., 2011).

Other important finding of this study is the fact that extrasynaptic AMPAR upregulation during development of persistent inflammatory pain was observed specifically in a subpopulation of SG neurons that exhibit intrinsic tonic firing properties, with no significant changes in the neurons that exhibit strong adaptation (transient neurons). This indicates that CFA alters extrasynaptic AMPARs in tonic firing SG neurons, whereas the pool of extrasynaptic AMPAR in the transient neurons is stable during the development of inflammatory pain and even during the persistent pain maintenance (Kopach et al., 2011). Taking into account similar patterns of extrasynaptic AMPAR expression in tonic and transient SG neurons under normal conditions (Kopach et al., 2011), these findings suggest different contribution of neuronal subtypes to the detection and/or transmission of nociceptive stimuli, resulting in different involvement in persistent inflammatory pain development and maintenance.

In conclusion, our study shows that CFA-induced peripheral inflammation upregulates extrasynaptic AMPARs specifically in tonic firing SG neurons during the development of inflammatory pain. These neurons may represent a specific population of neurons that carry out nociceptive inputs and contribute to spinal central sensitization during development and maintenance of persistent inflammatory pain. The upregulated extrasynaptic AMPARs in dorsal horn neurons during the inflammatory pain development might contribute to persistent pain hypersensitivities.

### Conflict of interest statement

The authors declare that the research was conducted in the absence of any commercial or financial relationships that could be construed as a potential conflict of interest.
